# Bioprospecting and Diversity of Yeast Producing Ethanol Isolated from Indonesia

**DOI:** 10.21315/tlsr2022.33.3.1

**Published:** 2022-09-30

**Authors:** Eny Ida Riyanti, Rafika Yuniawati, Edy Listanto

**Affiliations:** Indonesian Centre for Agricultural Biotechnology and Genetic Resources Research and Development, Jl. Tentara Pelajar No 3A, Bogor 16111, West Java, Indonesia

**Keywords:** Bioprospecting, Bioetanol, Yeast, Phylogeny, Indonesia, ITS Area

## Abstract

Bioethanol is considered the most environmentally friendly as renewable fuels. Indonesia has abundant microbe diversity which is potential for bioprospecting such as fermenting agents using agriculture product as raw materials for producing bioethanol. This study aims to isolate, characterise and molecular identify of 15 isolates of bioethanol-producing yeasts from various sources. Characterisation based on ethanol production, cell morphology and various substrate utilisation has been carried out. Molecular characterisation of 15 yeast isolates using tree sets of primers had been carried out. Amplification in the internal area of transcribe spacers (ITS) was successfully carried out with an amplitude of 400 bp–900 bp. Amplifiers in the D1/D2 26s rDNA domain are 250 bp. Amplification with ScerF2 and ScerR2 specific primers was carried out successfully and proved that there were two isolates which were not *Saccharomyces cerevisiae* analysis of yeast genetic diversity showed 12 yeast isolates classified as *S. cerevisiae* and the rest belonged to the genus *Clavispora*, *Candida* and *Kodamaea* (*Pichia*).

**Highlights**
- Fifteen ethanol producing yeast isolate have been identified from Indonesia.
- Fermented foods are a rich source of ethanol producing yeast.
- Isolate P (KBM) 10.5.1.4 is a potential new species as it has only 86.2% homology to kwon accession in the Genebank, *Saccharomyces cerevisiae* strain YR29.

## INTRODUCTION

Fossil fuels such as oil, coal and natural gas emit greenhouse gases (GHG) emission, caused air pollution and harm to the environment. Spikes in oil prices and concerning on the effects of climate change, have lead new urgency to the search for clean, renewable fuels (Selim *et al*. 2020; IRENA 2019). National efforts to reduce the need for fossil fuel by utilising alternative renewable energy is stated in the Republic of Indonesia Presidential Regulation (PERPRES) Number 5 of 2006 concerning national energy policy through the development of bio-based renewable energy. Biofuel is energy derived from living matter, especially plants. There are three types of biofuels bigest produced in the world: bioethanol, biodiesel and biogas. Bioethanol has potential to be used as fossil fuels substitution, as it can be mixed with gasoline called gasohol E-10, a mixture of pure gasoline with 10% pure bioethanol with an octane value of almost 92. The advantages of bioethanol include producing high energy, can reduce 12% of greenhouse gases, and are more environmentally friendly than biodiesel.

There are various ways of making biofuels, but they generally use mechanical, physical, chemical reactions, enzymatic reaction and heat to break down the starches, sugars, and other molecules in plants. The resulting products are then refined to produce a fuel that cars or other vehicles can use. Production of biofuel so far has used agricultural products as its basic material. Raw materials for bioethanol are sugar (sugar cane and sorghum juice) or carbohydrates (cassava, wheat, corn, sweet potatoes, soybeans and sorghum seeds) which is called as first generation of bioethanol (Tse *et al*. 2021; Salelign & Duraisamy 2021; Appiah-Nkansah *et al*. 2019). Second generation bioethanol utilises biomass in the form of non-food residues such as lignocellulosic waste (Fivga *et al*. 2019; Robak & Balcerek 2020). Fermentation is a series of reactions aided by microorganisms as biocatalysts, one of which is yeast. The type of yeast that is often used for alcohol production is *Saccharomyces cereviseae*. *S. cereviseae* is the oldest, most robust ethanol producer (Parapouli *et al*. 2020; Malairuang *et al*. 2020; Casas-Godoy *et al*. 2021)

Report on the isolation of ethanol producing yeast have been done from diverse sources such as fruit, soil and molases and others (Johansen *et al*. 2019; Phong *et al*. 2019; Riyanti *et al*. 2020; Kechkar *et al*. 2019). Yeast identification can be determined based on morphology and biochemistry but still need further identification to determine the type to be more accurate. Yeast identification methods with molecular approaches have high accuracy and can provide information to species level (Arastehfar *et al*. 2019). Ribosomal DNA (rDNA) is the genome encoding region for the ribosomal RNA component. The rDNA region is separated from one another by a barrier called a spacer (Schoch *et al*. 2012; O’Brien *et al*. 2005). The method commonly used to identify types of yeast isolates is by sequencing methods in the internal area of transcribe spacers (ITS) (Chanprasartsuk *et al*. 2013; Pramateftaki *et al*. 2000). Other molecular markers to ensure yeast identification are D1/D2 domains in 26s rDNA, almost all species of yeast data sequences in this area and the ITS area are available on an international database (Hesham *et al*. 2014).

Some yeast isolates derived from fermented foods from various cities in Indonesia were isolated and identified in Molecular Biology Laboratory, Centre for Research and Development of Biotechnology and Agricultural Genetic Resources (BB Biogen). This study aims to select yeast that produces high levels of bioethanol using high performance liquid chromatography (HPLC), morphological and biochemical characterisation and molecular identification or phylogenetic analysis of bioethanol-producing yeast by amplifying parts of the ITS region.

## MATERIALS AND METHODS

### Yeast Isolation, Purification and Screening for Ethanol Production

Yeast was isolated from various sources using yeast potato dextrose (YPD) agar medium containing yeast extract (10 g/L), bacto peptone (20 g/L) and glucose (20 g/L) following Cold Spring Harbour (CSH) Protocol with modification (2010). The material sample was suspended into sterile water followed by dilution to 10^−5^ in sterile water, and then 100 μL aliquot was spread cultured into YPD agar medium in triplicate. The culture was then incubated in 30°C for several days. Single colony grown in the YPD agar medium was then purified by streaking on to new YPD agar medium. Several pure cultures were chosen and cultured into 10 mL of YPD broth, and incubate at 30°C for 24 h, with 125 rpm shaking. Ethanol production was measured using High Performance Liquid Chromatography (HPLC) machine (Agilent 126 Infinity, International Equipment Trading Ltd, Illinois, USA). Strain producing ethanol was chosen for evaluation using different sugar sources.

### Carbon and Cellulose Utilisation

Three media with different carbon sources were tested, YPD containing glucose as carbon sources (20g/L) and yeast extract (10 g/L), bacto peptone (20 g/L) and glucose (20 g/L), YPX is YPD containing xylose (20 g/L), and YPGX containing glucose (20 g/L) and xylose (20 g/L) (Modified from CSH Protocol, 2010). For capability for degrading cellulose, medium CMC was used containing KH_2_PO_4_ (1 g/L), MgSO_4_ (0.5 g/L), NaCl (0.5 g/L), FeSO_4_ (0.01 g/L), 0.5 g NH_4_NO_3_ (1 g/L), MnSO_4_ (0.01 g/L) and CMC (10 g/L) (Lo *et al*. 2009). All medium was adjusted to pH 7.

### Cell Morphology

The chosen isolate was examined morphologically using light microscope with 1000× magnitude attached with image capture device. Cell measurement was done using hemocytometer with the calculation based on the comparison of grid size and volume.

### Fermentation Process

Fermentation proccess was carried out using batch system in Mac Cartney bottles. A total of 1 mL of overnight seed culture were put into 9 mL of YPD media for fermentation process. Fermentation was carried out for 24 h on YPD media at 30°C, 150 rpm. Observations were made every 24 h.

### Products Evaluation of Selected Isolate

Two chosen isolate with highes ethanol production was characterise based on ethanol yield. Ethanol yield was calculated based on dp/ds, when dp is ethanol production and ds is carbon used during the fermentation process as follows.


$$\text{Yp}/s=\frac{P}{\Delta S}$$

where *P* (product) is ethanol concentration, *s* (substrate), Δ*S* is the total glucose consumed where obtained from initial glucose level and *S* is the remaining glucose concentration in the end of fermentation.

### Sample Analysis Using HPLC

Sample was taken from the culture at 2 mL volume followed by centrifugation at 5,000 rpm, 4°C for 5 min. The cell biomass was used for biomass determination. Supernatant was then filtered using disposable 0.2 μL diameter filter and transferred into HPLC vial for analysis using HPLC (Agilent 126 Infinity, Agilent Technology) equipped with Refraction Index Detector (RID) detector (Agilent 126 Infinity, International Equipment Trading Ltd, Illinois, USA). Hi-plex H for carbohydrate collumn was used for the analysis using ddH_2_O as mobile phase and maintained at at 0.7 mL flow rate, and 60°C–70°C collumn temperature.

Filtrate was then analysed for ethanol production, sugars (glucose, xylose) content and side products (acetic acid, lactic acid and glycerol) content. Substrate were identified related to their peaks at various retention time which are glucose at 8.49 min, xylose for 9.01 min, ethanol for 18.96 min, acetic acid for 13.27 min, lactic acid for 11.27 min and glycerol for 11.62 min. The concentration of each compound was determined based on its peak area compared to series of standard’s peak area. Data on the ethanol and side product were analysed using F-test.

### Isolation of Genomic DNA

The DNA isolation process was carried out following the standard protocol of YeaStar^TM^ Genomic DNA Kit with protocol II (Thermo Fisher Scientific, Massachusetts, USA). The amount of 1.5 mL cell cultures were centrifuged at 5,000 rpm for 2 min, and supernatant was discarded. A total of 120 μL YD digestion buffer and 5 μL R-zymolyase was then added into pellets. The pellets were resuspended with vortex and incubated at 37°C for 40 min–60 min. Then, 120 μL of YD lysis buffer was added and mixed with vortex for 10 s to 20 s. The mixture was centrifuged at 10,000 rpm for 2 min. The supernatant was then transferred to the zymo-spin III column and re-centrifuged at 10,000 rpm for 1 min. Amount of 300 μL DNA washing buffer was added to the column and centrifuged at a speed of 10,000 rpm for 1 min. Then, 300 μL DNA washing buffer was added to the column and centrifuged at 10,000 rpm for 1 min. The zymo-spin III column was transferred into a sterile 1.5 mL centrifuge tube and ddH_2_O was added to the 60 μL column membrane. The column was incubated for 1 min at room temperature then centrifuged again at 10,000 rpm for 10 s. The liquid DNA was then collected in a 1.5 mL tube and added to 1 μL of RNAse A then homogenised. Several isolates need modification method during cell lysis. Qualitative DNA examination was performed with 1% agarose gel electrophoresis dyed with Etidium Bromide and visualised using UV translulimator (Sambrook & Russel 2001).

### DNA Quantity Measurement

The quantity of DNA was determined using NanoDrop™ 2000/2000c Spectrophotometer (Thermo Scientific, Waltham, Massachusetts, USA) according to its manual. The data obtained in the form of concentration (ng/μL) and DNA purity (A260/280 ratio).

### Amplification of 5.8S Gene Spacer

Three pair primers were used in this experiment, ITS1 (5′-TCC GTA GGT GAA CCT GCG G-3′) dan ITS4 (5′-TCC TCC GCT TAT TGA TAT GE-3′), primer for 26s rDNA region NL1 (5′-GCA TAT CAA TAA GCG GAG GAA AAG-3′) and LS2(5′-ATT CCC AAA CAA CTC GAC TC-3′) (Chanprasartsuk *et al*. 2013), and specific primers for *S. cerevisiae* ScerF2(5′-GCG CTT TAC ATT CAG ATC CCG AG-3′) dan ScerR2 (5′-TAA GTT GGT TGT CAG CAA GAT TG-3′) (Suranska *et al*. 2016). Amplification was done using PCR machine using program consisted of 30 cycles with an initial temperature of 95°C for 10 s, a denaturation temperature of 95°C for 30 s, annealing temperature at 59°C for 1 min, an extension temperature of 72°C for 1 min, and an elongation temperature of 72°C for 10 min. Several isolates used modified optimum temperature and duration in the annealing step. Visualisation of the amplicon was done with 1% agarose gel.

### Gene Sequencing and Bioinformatics Analysis

Sequencing was done in First Base Company, Singapore. Sequence analysis was performed using BioEdit software. The sequence homology was anlysed using BLASTn programme (Altschul *et al*. 1990; Boratyn *et al*. 2013). Phylogenetic analysis using the MEGA 7.0 program with the neighbour-joining (NJ) method (Kumar *et al*. 2016).

## RESULTS

### Screening Yeast Isolates for Bioethanol Production

Fifteen pure isolates were obtained from the isolation process. Data of ethanol and side products analysis was presented in Table 1.

In this screening process, the isolates produced ethanol at 0.8 up to 2.61 g/L, which most of the isolate produced side products such as acetic acid, lactic acid and glycerol. Using 24 h fermentation process, glucose was detected in the culture, which mean the fermentation process need longer than 24 h.

### Growth Analysis on Various Carbon Sources

The growth of 15 isolates in four media with different carbon sources were presented in Table 2. It shows that all isolates can grow in YPG, YPX and YPGX media with different growth rate observed from the colony formation. Meanwhile, all isolates could not grow in CMC media which indicated that 15 isolates did not have cellulolytic activity. Isolates grown in media containing glucose can grow faster compared to media containing xylose and mixture glucose and xylose.

**Figure 1:**
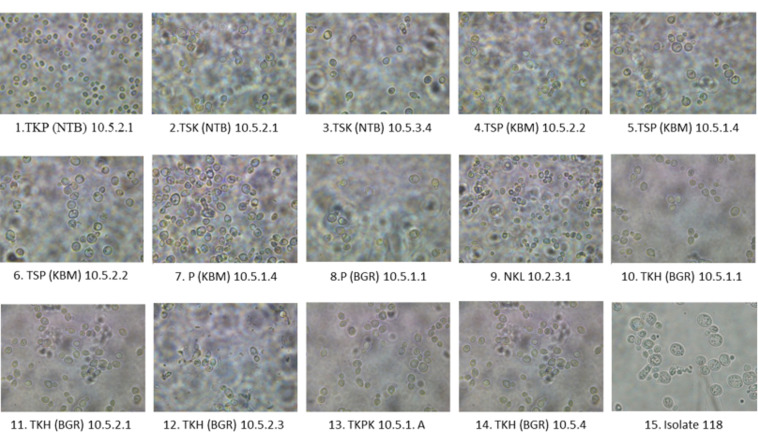
Cell morphology of 15 yeast isolates using light microscope (1000× magnitude).

Under light microscope, cell morphology of 15 isolates were haploid with oval to round shape cells as displayed in Fig. 1.

### Yield Observation

Two isolates with highest ethanol product were compared based on their yields. Table 3 shows the yield comparison between isolates NKL 10.5.2.3 and TKH (BGR) 10.5.2.1. Two isolates produced relatively the same amount of ethanol, but it produced different side product and glucose consumed. Based on the glucose consumed during the fermentation process, isolate NKL 10.5.2.3 gave higher yield compared to TKH(BGR)10.5.2.1.

### Molecular Identification of Yeast Isolates

**Figure 2:**
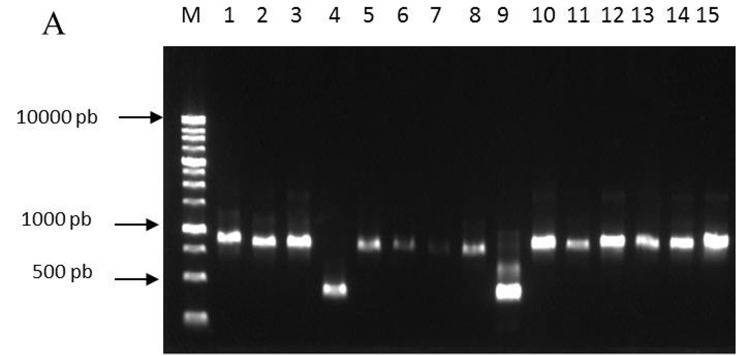

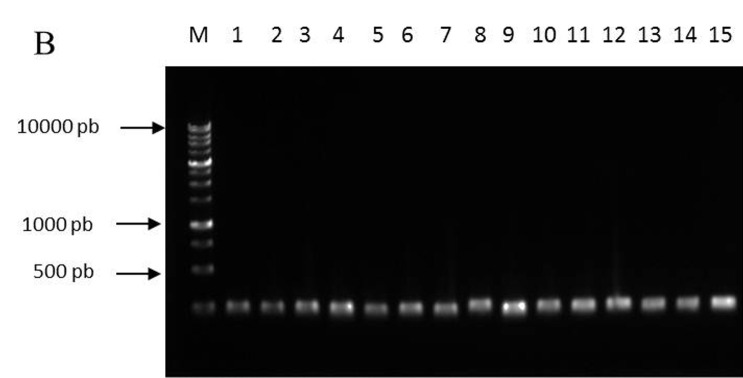

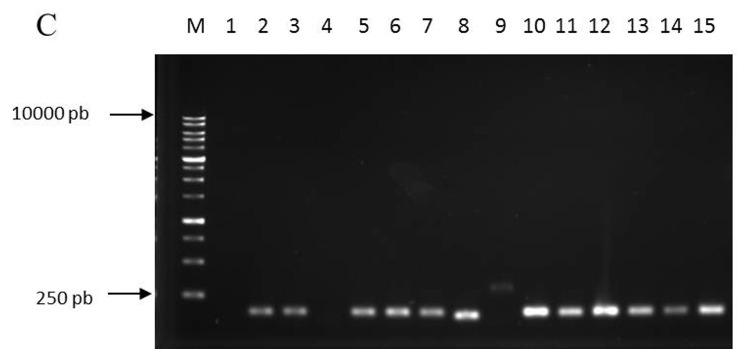
DNA fragment amplification on 1% agarose. M = 1 kb Ladder DNA and 1–15 = isolate number 1–15. (A) Amplicon in the ITS area with ITS1 and ITS4 primers, (B) amplicon in the D1/D2 domain in the 26s rDNA gene with NL1 and LS2R primers and (C) amplicon with ScerF2 and ScerR2 specific primers.

DNA concentrations of 15 yeast isolates varied ranging from 10.4 ng/μL to 42.8 ng/μL. The amplification profile in the ITS area was around ± 900 bp for all isolate, except isolate number 4 [TKP (KBM) 10.5.2.2] and number 9 (NKL 10.5.2.3) which is around ± 400 bp (Fig. 2A). Amplification of the D1/D2 domain shown in Fig. 2B was around ± 250 bp. Amplification using specific primer for *S. cerevisiae* ScerF2 and ScerR2 produced amplicon around 250 bp. Using this primer set, two isolates [lane 1 TKB (NTB) 10.5.2.1 and lane 4, TKP (KBM) 10.5.2.2] did not produced expected amplicon (Fig. 2C). Suranska et al. (2016) stated that the genus *Saccharomyces* has an amplicon size of 150 bp. While isolate number 9 (NKL 10.5.2.3) has a length of about 300 bp with a thin band.

### Phylogeny Analysis

Table 4 describes the homology level of yeast isolates with other sequences of alignment results. Nine isolates had the maximum identity value obtained is more than 98% and there are six isolates that have a maximum identity value in the range of 86%–97%. Expectation-value (e-value) of all isolates is 0.0 except isolates NKL 10.5.2.3 which is 4e-176. A total of 13 isolates were high homology to *S. cerevisiae* with a maximum identity value in the range of 86%–99%. Isolate NTB 10.5.2.1 belongs to the genus *Candida* with a maximum identity of 94%. Isolate KBM 10.5.2.2 belongs to the genus *Kodamaea (Pichia*) with a maximum identity of 99%. Isolate NKL 10.5.2.3 belongs to the genus *Clavispora* with a maximum identity value of 95%, but this alignment has a bad e-value of 4e-176.

**Figure 3:**
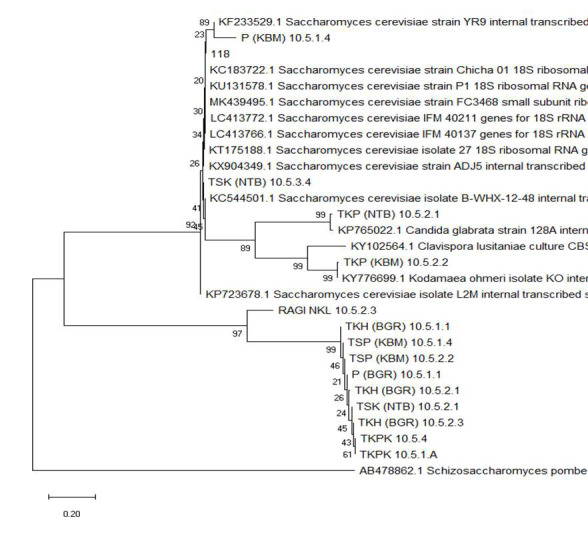
Yeast phylogenetic analysis dendogram based on ITS sequencing.

Fig. 3 shows the phylogenetic tree constructed using the ITS regional sequence. It shows that there are four genera belonging to the yeast with one species as an outgroup, *Schizosaccharomyces pombe*. *Candida* genus group [TKP (NTB) 10.5.2.1] with a boostrap value of 99% and have a close kinship with *Clavispora* and *Kodama* with a boostrap value of 89%. The *Kodamaea* group [TKP (KBM) 10.5.2.2] with a high boostrap value of 99% and has a close kinship with the genus *Clavispora*. Isolate TSK(NTB) 10.5.3.4 has a close relationship with the genus *Saccharomyces* with a low boostrap value of 41%.

TSP (KBM) 10.5.1.4 isolate is closely related to the genus *Saccharomyces* with a boostrap value of 89% and has another kinship with isolate 118 with a small boostrap value of 23%. Isolate TKPK are in tree branches with a boostrap value of 61%. This indicates that isolate TKPK have a close relationship, the similarity in the resulting pattern as both isolates isolated from the same food source of fermented black sticky rice. TKPK isolates are related to TKH (BGR) 10.5.2.3 because the source of insulation is the same, sticky rice tape. TKH (BGR) 10.5.1.1, TSP (KBM) 10.5.1.4, TSP (KBM) 10.5.2.2, P (BGR) 10.5.1.1, TKH (BGR) 10.5.2.1, TSK (NTB) 10.5.2.1, TKH ( BGR) 10.5.2.3, TKPK 10.5.1.A, and TKPK 10.5.4 are in a group that has a close kinship with NKL 10.5.2.3 with a booostrap value of 97% which belongs to the genus *Saccharomyces*.

## DISCUSSION

Fifteen yeast isolates had been characterised for the ability to produce ethanol (Table 1), and capability using various sugars sources (Table 3). All the isolate grow on glucose medium and mixture sugars glucose and xylose, however slightly growing on sole xylose medium (Table 2). This could indicate in the fermentation process that the isolates uptaken glucose, and then xylose after the glucose depleted. However, it could also mean that the isolate could co-fermenting glucose and xylose with different rate. Morphological examination under microscope shows that all of them was haploid cell, with diverse shape from oval to circular. It was reported that the diversity of yeast species in different habitat is determined by its capability of utilising different carbon source and its nutritional selectivity as it exhibits their great specialisation (Mohd Azhar *et al*. 2017).

The highest ethanol concentration of 2.61 g/L produced by NKL 10.5.2.3 and TKH (BGR) 10.5.2.1, even though not significantly different statistically. The highest isolates were able to produce bioethanol with the same level of 2.61 g/L but the isolate TKH (BGR) 10.5.2.1 changed less sugar in the media into fermented product compounds, such as TKH isolate (BGR) 10.5.2.1 only able to convert as much as 13.47% glucose (Table 1). This value is smaller than NKL 10.5.2.3 isolate which was able to convert 13.51% glucose. The low concentration of ethanol in this result was due to low sugar concentration in the medium (20 g/L glucose) and short period of fermentation process (24 h fermentation time) as sugar remained in the medium after fermentation process (Table 2). Muin *et al*. (2015) stated ethanol levels will continue to rise until the 72nd hour but the ethanol and CO_2_ levels produced can inhibit ethanol production in the next hour. In addition, according to Rahmana *et al*. (2016), at the 72nd hour yeast is still in a stationary state so that most likely the sugar will be consumed optimally. The fermentation process will slow down when the concentration of ethanol produced is around 12%–15% because yeast cells are only tolerant at certain ethanol concentrations.

Using 20 g/L glucose in the medium, isolates could give higher ethanol product in prolong time as the glucose remained 0.69 g/L and 0.63 g/L, respectively in the media. Moreover, it is predicted that all the isolates could produce higher products if the medium contain higher carbon source concentration, longer fermentation process and optimum condition after bioprocess optimisation (Table 2).

Capability of using wide range carbon sources including hexose and pentose is very important for industrial purpoces. The capability of isolate using pentose is very important for further application using lignocellulosic hydrolysate which contained mixture hexose and pentose carbon. Research on second generation of ethanol production is still hot topic for lowering cost production from using cheap raw material such as agriculture waste containing lignocellulosic materials. However, lignocellulosic hydrolysate is recalcitrant for microbial fermentation due to sugars mixture pentose and hexose sugars and other compounds. This pentose sugars and other inhibitors act as inhibitor for industrial yeast so far (Robak & Balcerek 2020). Based on the screening on Table 2, the capability of growing in medium with pentose (xylose) is promising for this isolate to be used in lignocellulosic hydrolysate medium for second generation of ethanol production. In this result, all isolate is capable using glucose and xylose, however less growth on xylose compared to on glucose or mixture glucose and xylose observed.

In this result, all isolate tested could not grow on the cellulose medium, this condition indicated that yeast isolates did not have cellulolytic activity. Yeast isolates that have been tested do not prove the existence of cellulolytic activity so that if large-scale biethanol production will be carried out with a source of agricultural biomass material it is necessary to have pretreatment.

Two highest ethanol production (2.61 g/L, using 20 g glucose for 24 h fermentation process) had been identified, isolate NKL 10.5.2.3 and TKH (BGR) 10.5.2.1. However, isolate NKL 10.5.2.3 have higher yield (13.51%) compared to TKH (BGR) 10.5.2.1 (13.47%). The two isolates produced different side product. Both isolates produced glycerol. TKH (BGR) isolate 10.5.2.1 did not produce lactic acid while isolate NKL 10.5.2.3 did not produce acetic acid (Table 3). This propertis might give useful information for further fermentation process and genetic improvement for these isolates in the future.

The low yield of ethanol obtained in this study might due to the low concentration of carbon and the length of fermentation process. Batch culture system with *S. cerevisiae* was used for ethanol fermentation with glucose concentrations ranging 10 g/L–260 g/L, as a result, 0.2 g/L–7.0 g/L biomass and 5.1 g/L–115.0 g/L ethanol were obtained. The maximum yield of ethanol by yeast is theoretically about 51% or 0.51 g ethanol/g glucose. High ethanol concentration and ethanol yield could be achieved by the fed-batch cultures with total glucose concentrations up to 260 g/L (Chang *et al*. 2018).

Sequence alignment in the ITS area showed 13 isolates of *S. cerevisiae* and other isolates of *Clavispora, Kodamae (Pichia*) and *Candida*. Isolate NKL 10.5.2.3 according to homology analysis are *Clavispora lusitaniae* and isolate TKH (BGR) 10.5.2.1 is *S. cerevisiae*.

Generally, the maximum identity value obtained is more than 98% and there are six isolates that have a maximum identity value in the range of 86%–97% (Table 3). A high maximum identity value indicates a greater degree of homology between sequences. In addition, the e-value is also considered. The e-value is a value that states the level of statistical probability to have similarities with the tested sequence or commonly referred to as the percentage error. The smaller the e-value indicates the level of error and the higher the level of homology. The maximum identity value listed in the BLAST results is influenced by other parameters displayed in the BLAST (Claverie & Notredame 2006).

E-value of all isolates is 0.0 except isolates NKL 10.5.2.3 which is 4e-176 (Table 3). This indicates that NKL 10.5.2.3 with the results of the alignment has a fairly large error rate (Nugraha *et al*. 2014). A total of 13 isolates were parallel to *S. cerevisiae* with a maximum identity value in the range of 86%–99%. Isolate TKP (NTB) 10.5.2.1 belongs to the genus *Candida* with a maximum identity of 94%. Isolate TKP (KBM) 10.5.2.2 belongs to the genus *Kodamaea (Pichia)* with a maximum identity of 99%. Isolate NKL 10.5.2.3 belongs to the genus *Clavispora* with a maximum identity value of 95%, but this alignment has a bad e-value. Sequences with a maximum identity value <95% are labelled as unknown organisms (Rosa *et al*. 2010). Meanwhile, sequences with a maximum identity value more than 97% indicate identical species. Another theory states, the maximum identity value in the range 93%–97% represents the same genus level with the possibility of different species levels. Maximum identity values in the range 82%–93% indicate novelty at the genus level (Hagstrom *et al*. 2000). In this regard, isolate P (KBM) 10.5.1.4 has only 86.2% homology to *S. cerevisiae* strain YR29. Fig. 3 shows that this isolate has far distance to other identified *S. cerevisiae*. Therefore, this isolate could be a candidate for new species, but need further investigation.

Phylogenetic analysis in this study uses the neighbour-joining reconstruction method with a boostrap evaluation of 1,000 repetitions. Evaluation of phylogenetic trees using boostrap is carried out to determine the level of trust in phylogenetic trees. Determination of phylogenetic reliability of reconstructed trees is based on boostrap values, if the value is more than 70% of tree branches has high stability (Osawa *et al*. 2004).

Relationship analysis of test sequences with BLAST result sequences must include outgroup groups for apomorphic and plesiomorphic character polarisation. This study uses *Schizosaccharomycess pombe* as an outgroup species.

*S. cereviseae* and *Pichia stipitis* are common yeast used for bioethanol production. *S. cereviseae* is unable converting pentose sugar which is 45% content of the lignocellulosichydrolysate (Nijland & Driessen 2020). Meanwhile, *Pichia stipitis* has great potential to ferment hexose and pentose sugars such as xylose to be used as ethanol with high levels (Ishizaki & Hasumi 2013). Kinship between species is known by phylogenetic analysis which is a combination of molecular techniques and statistics. It has been known that the results of BLAST showed four large genera that correspond to the test sequences namely *Saccharomyces, Clavispora, Kodamae (Pichia*) and *Candida*. Yeast isolated from all indigenous sub-Saharan African fermented food and beverages was also reported by Johansen *et al*. (2019) which being the predominant by *S. cerevisiae*.

## CONCLUSION

In conclusion, two candidate isolates of yeast, NKL 10.5.2.3 (*Clavispora lusitaniae* culture CBS:5299) and TKH (BGR) 10.5.2.1(*S. cerevisiae* isolate L2M), were promising for further investigation for ethanol production since both recorded higher ethanol production compared to the other isolates. Besides, isolate P (KBM) 10.5.1.4 needs further investigation as a candidate for new species.

## Figures and Tables

**Table 1:** Screening for ethanol production and side products of the isolates.

No.	Isolate code^*^	Ethanol (g/L)^nt^	Acetic acid (g/L)^nt^	Lactic acid (g/L)^nt^	Glycerol (g/L)^nt^	Remaining glucose (g/L)^nt^
1	TKP (NTB) 10.5.2.1	2.54	0.00	0.08	1.00	0.80
2	TSK (NTB) 10.5.2.1	2.39	0.79	0.00	1.50	0.63
3	TSK (NTB) 10.5.3.4	2.42	0.73	0.00	1.50	0.68
4	TKP (KBM) 10.5.2.2	1.79	0.00	0.61	1.30	0.72
5	TSP (KBM) 10.5.1.4	2.40	0.00	0.03	1.60	0.65
6	TSP (KBM) 10.5.2.2	0.75	0.00	0.01	1.70	0.72
7	P (KBM) 10.5.1.4	2.54	0.73	0.00	0.60	0.59
8	P (BGR) 10.5.1.1	0.80	0.01	0.22	1.00	0.71
9	NKL 10.5.2.3	2.61	0.00	0.63	0.90	0.69
10	TKH (BGR) 10.5.1.1	2.50	1.09	0.07	1.00	0.63
11	TKH (BGR) 10.5.2.1	2.61	0.35	0.00	0.70	0.63
12	TKH (BGR) 10.5.2.3	2.39	0.77	0.11	0.90	0.59
13	TKPK 10.5.1.A	2.41	0.91	0.10	1.30	0.72
14	TKPK 10.5.4	1.96	0.00	0.17	1.20	0.77
15	Isolate 118	2.31	0.81	0.12	1.50	0.57

*Note*: nt = Not significant different, F-distribution: α = 5%

**Table 2:** The growth of yeast isolates on different carbon sources.

No.	Isolate code	Source/location	Medium			

			YPG	YPX	YPGX	CMC
1	TKP (NTB) 10.5.2.1	Fermented glutinouse white rice from west Nusa Tenggara	+++	+	++	−
2	TSK (NTB) 10.5.2.1	Fermented cassava/West Nusa Tenggara	+++	+	++	−
3	TSK (NTB) 10.5.3.4	Fermented cassava/Kebumen	+++	+	++	−
4	TKP (KBM) 10.5.2.2	Fermented glutinious white rice/ Kebumen	+++	+	++	−
5	TSP (KBM) 10.5.1.4	Fermented cassava/Kebumen	+++	+	++	−
6	TSP (KBM) 10.5.2.2	Fermented cassava/Kebumen	+++	+	++	−
7	P (KBM) 10.5.1.4	Fermented black fermented casava/ Kebumen	+++	+	++	−
8	P (BGR) 10.5.1.1	Fermented casava/Bogor	+++	+	++	−
9	NKL 10.5.2.3	Inoculum for casava tapai	+++	+	++	−
10	TKH (BGR) 10.5.1.1	Fermented glutinious black rice/ Bogor	+++	+	++	−
11	TKH (BGR) 10.5.2.1	Fermented glutinious black rice/ Bogor	+++	+	++	−
12	TKH (BGR) 10.5.2.3	Fermented black glutinious rice/ Bogor	+++	+	++	−
13	TKPK 10.5.1. A	Fermented glutinious black rice/ Bogor	+++	+	++	−
14	TKPK 10.5.4	Fermented glutinious black rice/ Bogor	+++	+	++	−
15	Isolate 118	Molases waste, Yogyakarta	+++	+	++	−

*Notes*: (−) not grow, (+) grow, (++) grow better, (+++) very well grown, YPG (glucose medium), YPX (xylose medium), YPGX (glucose xylose medium) and CMC (Carboxymethylcellulose medium).

**Table 3:** Yield comparison of two highest ethanol producer.

Products (g/L)	NKL 10.5.2.3		TKH (BGR) 10.5.2.1	

	Product concentration (g/L)	Yield (%)	Product concentration (g/L)	Yield (%)
Ethanol	2.61	13.51	2.61	13.47
Lactic acid	0.63	3.26	0.00	0.00
Acetic acid	0.00	0.00	0.35	1.80
Glycerol	0.90	4.66	0.70	3.61
Remaining glucose	0.69	-	0.63	-

*Note:* YPD medium containing 20 g/L glucose.

**Table 4:** BLASTn sequence analysis of ITS region.

No.	Isolate codes	BLAST results	Identity value (%)	e-value	Accession number
1	TKP (NTB) 10.5.2.1	*Candida glabrata* strain 128A	94.55	0.0	KP765022.1
2	TSK (NTB) 10.5.2.1	*S. cerevisiae isolates 27*	99.64	0.0	KT175188.1
3	TSK (NTB) 10.5.3.4	*Saccharomyces cerevisiae isolates* B-WHX-12-48	99.87	0.0	KC544501.1
4	TKP (KBM) 10.5.2.2	*Kodamaea (Pichia) ohmeri isolate* KO	99.44	0.0	KY776699.1
5	TSP (KBM) 10.5.1.4	*S. cerevisiae* strain P1	98.46	0.0	KU131578.1
6	TSP (KBM) 10.5.2.2	*S. cerevisiae* IFM 40137	95.57	0.0	KY105166.1
7	P (KBM) 10.5.1.4	*S. cerevisiae* strain YR29	86.22	0.0	KF233529.1
8	P (BGR) 10.5.1.1	*S. cerevisiae* strain ADJ5	96.85	0.0	KX904349.1
9	NKL 10.5.2.3	*Clavispora lusitaniae culture* CBS:5299	95.92	4e-176	KY102564.1
10	TKH (BGR) 10.5.1.1	*S. cerevisiae* IFM 40211	94.98	0.0	LC413772.1
11	TKH (BGR) 10.5.2.1	*S. cerevisiae isolate* L2M	97.92	0.0	KP723678.1
12	TKH (BGR) 10.5.2.3	*S. cerevisiae* strain FC3468	99.17	0.0	MK439495.1
13	TKPK 10.5.1.A	*S. cerevisiae isolates 27*	99.64	0.0	KT175188.1
14	TKPK 10.5.4	*S. cerevisiae* strain FC3468	99.52	0.0	MK439495.1
15	Isolate 118	*S. cerevisiae* strain Chica 01	99.29	0.0	KC183722.1
